# Pancreatic sarcoidosis: A case report and literature review

**DOI:** 10.1097/MD.0000000000041957

**Published:** 2025-03-28

**Authors:** Rui Li, Chenghua Zhu, Nan Gao, Qian Liu, Qiang Du

**Affiliations:** a Department of Nanjing Medical University Second Affiliated Hospital, Jiangsu, China; b Department of Respiratory Medicine, Pukou Hospital of Chinese Medicine affiliated to China Pharmaceutical University, Nanjing, Jiangsu, China.

**Keywords:** gastrointestinal symptoms, lymph nodes, pancreas, sarcoidosis

## Abstract

**Rationale:**

Sarcoidosis is a multisystem disease of unknown etiology that usually involves the lungs and hilar lymph nodes and rarely involves the digestive system other than the liver.

**Patient concerns:**

A woman with no medical history presented with 2 pulmonary nodules and a pancreatic mass on chest computed tomography (CT) because of sputum production and shortness of breath.

**Diagnoses:**

Ultrasound bronchoscopy and pathology showed pulmonary sarcoidosis.

**Interventions:**

Oral prednisone acetate.

**Outcomes:**

In the following year of follow-up, the pancreatic, lung and mediastinal lesions were significantly reduced.

**Lessons:**

Corticosteroid therapy may therefore constitute a different kind of diagnosis.

## 1. Introduction

Sarcoidosis is a multisystem non-caseous granulomatous disease that is usually caused by a combination of environmental and genetic factors that lead to the accumulation of CD4+T cells and mononuclear macrophages at the site of inflammation, promoting the formation of granulomas.^[1,2]^ The lungs and lung hilum, lymph nodes, skin, eyes and liver are the most common sites of involvement^[3]^; the pancreas is rarely involved, occurring in only 1% to 6% of patients with systemic sarcoidosis.^[4]^ The first case was described at autopsy in 1937. Here we present a case of pancreatic sarcoidosis without abdominal symptoms and review the relevant literature.

## 2. Case/case series presentation

**Figure 1.**
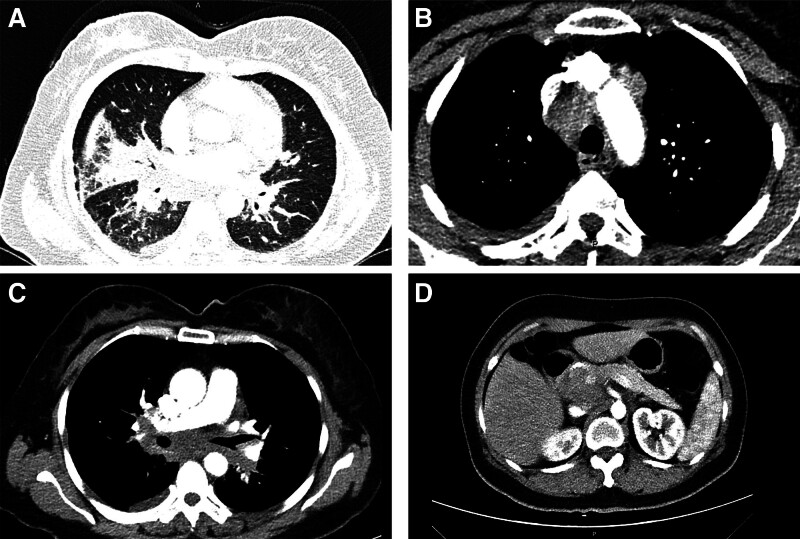
Enhanced chest computed tomography scan. Scattered nodules in both lungs and atelectasis (A), enlarged lymph nodes in groups 4R (B) and 7 (C), pancreatic mass (D).

A 56-year-old Asian woman with cough and sputum accompanied by asthma was treated at a local hospital for 4 months, and her symptoms did not improve significantly after anti-infective treatment. One month later, she was referred to Nanjing Yifu Hospital for a chest enhanced computed tomography (CT) scan (Fig. 1), which showed many swollen lymph nodes in the retroperitoneum and mediastinum, as well as a space in the head of the pancreas. She later visited our hospital for further management. Throughout the illness, the patient had no nausea, vomiting, abdominal pain or diarrhea, and no pruritus or yellowing of the skin. There was no history of tobacco or alcohol consumption. All tumor markers and immune indices were normal except for angiotensin-converting enzyme (121.70 U/L, reference ranges: 5.00–52.00 U/L).

**Figure 2.**
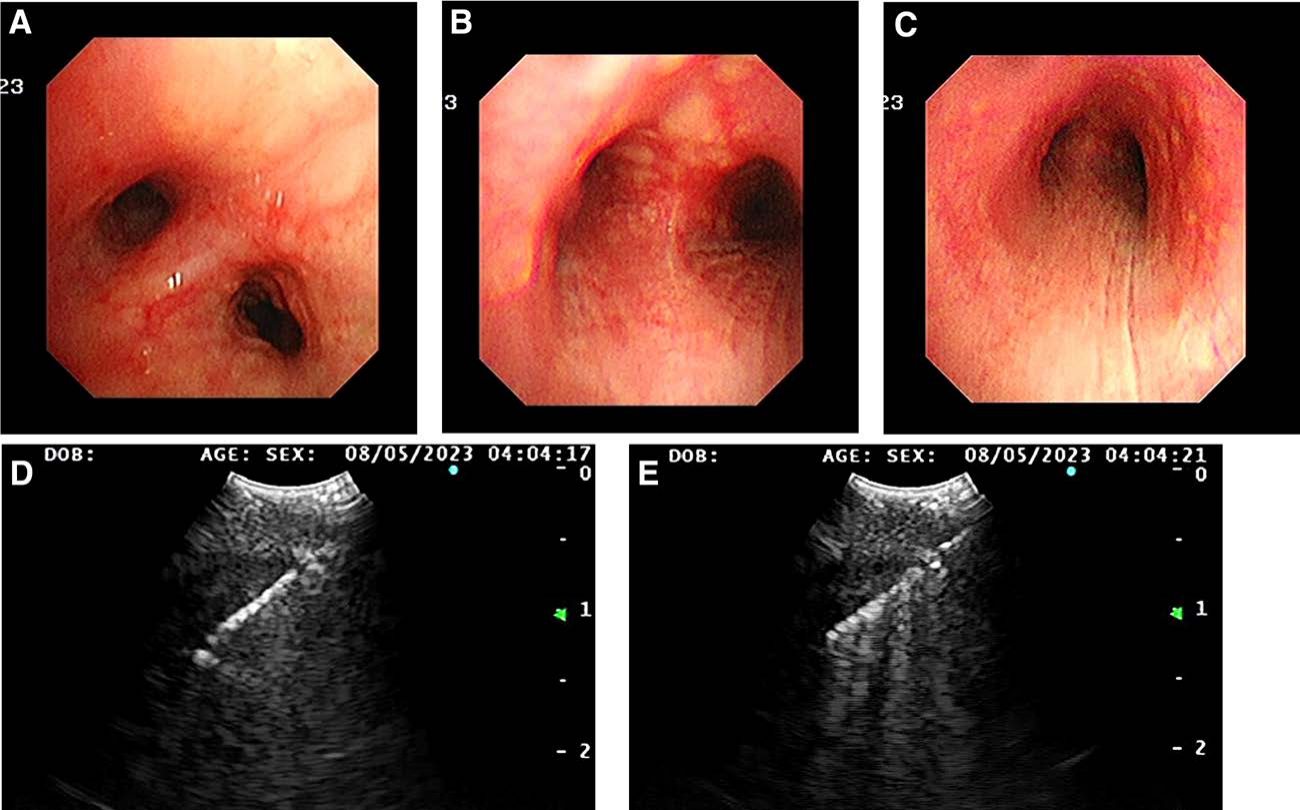
Bronchoscopy and EBUS-TBNA. Scattered nodular elevations in trachea and bronchi (A–C); enlarged lymph nodes in groups 4R and 7 (D and E). EBUS-TBNA = endobronchial ultrasound guided transbronchial needle aspiration.

**Figure 3.**
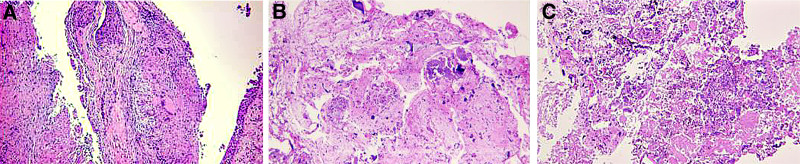
Bronchial mucosal and lymph node pathology. Epithelioid granuloma formation was seen in the bronchial subepithelial mesenchyme (A), 7 lymph nodes showed coagulative necrosis, cartilaginous tissue and scattered bronchial mucosal epithelium (B), 4R lymph nodes showed a small amount of cartilage and scattered bronchial mucosa with hemorrhage, and fibrinoid exudate (C).

**Figure 4.**
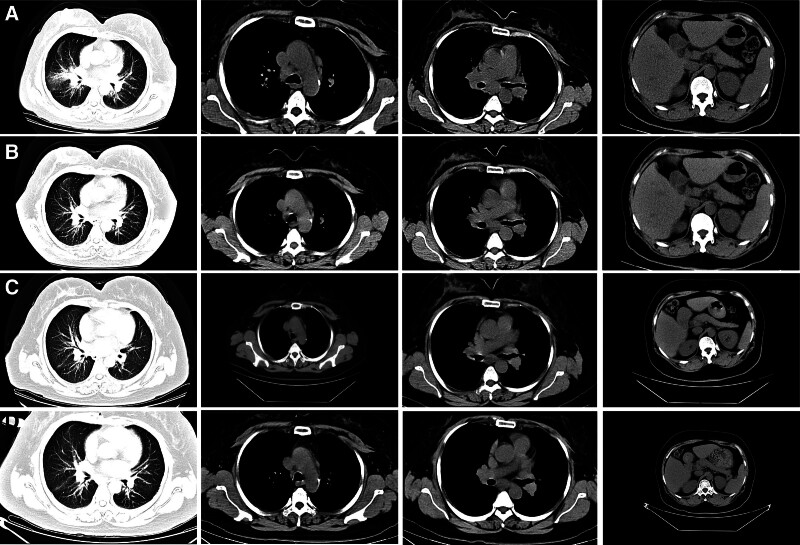
Nearly 1 yr follow-up: (A) September 7, 2023; (B) November 28, 2023; (C) February 27, 2024; (D) May 6, 2024.

In order to provide further clarification regarding the nature of the enlarged hilar lymph nodes, a bronchoscopy was performed, which revealed scattered nodular eminence in the trachea and bronchus, and extensive and mild stenosis of the lumen (Fig. 2A–C). Furthermore, ultrasonic bronchoscopy detected abnormal signals in 4R and 7 groups of lymph nodes (Fig. 2D and E). However, no clear tumor cells were found on the liquid-based smear of flushing fluid. The present study demonstrates that acute and chronic inflammation of the mucous membrane of the trachea (anterior segment of the upper right lobe and basal part of the lower right lobe) accompanied by erosion results in epithelioid granuloma formation in the subepithelial mesenchyme (Fig. 3A). A significant quantity of coagulated necrosis, cartilage tissue and scattered bronchial mucosa epithelium were identified in the “Group 7 lymph nodes” (Fig. 3B). In the “4R lymph nodes” (Fig. 3C), there were minimal quantities of cartilage, accompanied by sporadic bronchial mucosa exhibiting bleeding and cellulose-like exudates. As fungal infection, tuberculosis and other diseases can also lead to pulmonary granuloma, it was deemed necessary to improve the PPD test, tuberculin sputum smear, T-spot, Xpert and other tests in order to exclude the possibility of other granulomatous diseases. The results of these tests led us to rule out the possibility of pulmonary tuberculosis. Pulmonary fungal disease is predominantly a secondary infection, and the patient has no immunodeficiency disease or long-term use of immunosuppressants. Furthermore, there are no abnormalities in blood routine and sputum smear, and stable vital signs. In conjunction with bronchoscopy, the presence of fungal infection is not considered to be a priority at this time. Following the identification of sarcoidosis in the lung region during bronchoscopy, and despite the subsequent revelation of pancreatic head cancer by enhanced abdominal CT, the hypothesis that the spatial domain occupied by the pancreatic head and its enlarged lymph nodes is that of sarcoidosis involving the pancreas remains to be substantiated. In order to avoid a misdiagnosis, it was recommended that the patient undergo an endoscopic ultrasound puncture for the purpose of obtaining further confirmation. However, this was declined and instead, prednisolone acetate 30 mg/d was administered. During the 1-year follow-up period, the mass in the head of the pancreas and the mediastinal lymph nodes gradually decreased in size, the maximum length of the mediastinal lymph nodes decreased, and the number of nodules in both lungs decreased (Fig. 4).

## 3. Discussion

Regional and ethnic variations in the incidence and prevalence of sarcoidosis have been identified. The highest frequency of cases is observed in Sweden (160 per 100,000 people), followed by Canada (143 per 100,000 people). In contrast, Asians exhibit a comparatively low prevalence of the condition. A comparison of African Americans with Caucasian, Hispanic and Asian populations reveals that the incidence and prevalence rates for the former group are higher.^[5,6]^ Despite the ambiguity surrounding the precise etiology of sarcoidosis, Mycobacterium and Propionibacterium acnes are considered potential causative agents.^[7–9]^ These microorganisms have been observed to induce an amplified immune response from T cells and mononuclear macrophages, thereby fostering the development of nodular granulomas.^[2,3,10]^ The predominant cell type at the active regions of illnesses is T lymphocytes, particularly CD4+T helper (TH1) cells, which secrete cytokines and chemokines such as interleukin (IL)-2, tumor necrosis factor-α, interferon-γ, and transforming growth factor-β. Furthermore, the process of T cell activation has been observed to stimulate the formation of granulomas via the actions of IL-12, IL-15, and monocyte colony-stimulating factor, which are generated from macrophages.^[1,2]^ Research has indicated that MHC-2 gene variation is associated with the susceptibility, phenotype and prognosis of patients with sarcoidosis.^[3]^ Nevertheless, the question of whether other genes have an impact on sarcoidosis, and the manner in which they might do so, remains unresolved.

Sarcoidosis is a systemic disease that has the capacity to affect all systems of the body, including the lungs, lymph nodes, skin, heart, liver, musculoskeletal system, eyes, and other organs, but rarely the pancreas.^[3]^ A review of cases of sarcoidosis involving the pancreas was conducted by Martin Wijkstrom prior to 2005,^[11]^ following this review, a summary of cases after 2005 was compiled, of which 25 exhibited gastrointestinal symptoms^[11–34]^ (Table 1) and 9 did not^[4,35–42]^ (Table 2). Pancreatic sarcoidosis, characterized by the infiltration of pancreatic tissue or obstruction of the bile duct, may present with symptoms of pancreatitis or pancreatic cancer,^[12]^ Patients with pancreatitis may present with abdominal pain, nausea, vomiting, hypercalcemia, elevated serum amylase, etc. Abdominal CT may reveal signs of inflammation and granulomas in the pancreas and the area around it (pancreatic inflammation). Magnetic resonance cholangiopancreatography may show dilatation of the bile duct and the pancreatic duct.^[13–15,26,27,29,30,33]^ The presence of symptoms such as abdominal pain, skin discoloration, atrophy, and other indications of pancreatic cancer may be observed in cases where the pancreatic granuloma is sufficiently large to form a palpable mass. A CT scan of the abdomen may reveal peripancreatic or retroperitoneal lymph node enlargement.^[12,16–18,20–24,28,32,34]^ Furthermore, research has indicated that sarcoidosis has the potential to elevate the risk of developing various forms of cancer, including, but not limited to, lung, stomach, small intestine, liver, melanoma and nonmelanoma skin cancer, non-Hodgkin lymphoma, and leukaemia.^[43]^

**Table 1:** Pancreatic sarcoidosis with digestive symptoms.

Year	Author	Patient age/sex	Symptomatic	History of sarcoidosis	Diagnosis/treatment
2006	Cacares^[12]^	60 M	Jaundice, abdominal pain, wasting	No	Pancreaticoduodenect-omy
2006	Gupta^[13]^	52 F	Abdominal pain, nausea, vomiting	No	Transbronchial biopsies
2006	Pohlmann^[14]^	55	Abdominal pain	No	Lymph node biopsy
2007	Shukla^[15]^	54 M	Fatigue, loss of appetite, weight loss	Yes	CT scan guided pancreatic mass biopsy
2007	Harder^[16]^	71 M	Epigastric pain, fever, fatigue	No	Pancreaticoduodenect-omy
2008	Wellner^[17]^	68 F	Bloating, vomiting, exertional dyspnea	No	Frozen section
2010	Wijkstrom^[11]^	49 F	Abdominal pain, skin itching, anorexia, emaciation	No	Diagnostic laparoscopy
2011	Beridze^[18]^	29 M	Abdominal pain, jaundice	No	Whipple procedure
2011	Schauer^[19]^	29 M	Abdominal pain, jaundice	No	Whipple procedure and biopsy
2013	Mayne^[20]^	52 M	Jaundice, back pain, weight loss	No	Whipple procedure
2014	Zhang^[21]^	47 M	abdominal pain	No	Lymph node biopsy
2015	Khangura^[22]^	61 F	Abdominal pain, jaundice	No	Lymph node biopsy
2015	Hammen^[23]^	45 M	Abdominal pain, vomiting, jaundice, weight loss	No	Sonography-guided liver biopsy
2015	Storm^[24]^	32 F	Vomiting, abdominal pain, weight loss	No	Lymph node biopsy
2016	Hong^[25]^	41 M	Anorexia, weight loss	No	Biopsy
2016	Yao^[26]^	70 M	Diarrhea, weight loss	No	EUS-FNA
2017	Bihun^[27]^	61 F	Weight loss, scleral jaundice, abdominal pain, back pain	Yes	Pancreaticoduodenect-omy and needle biopsy
2017	Mony^[28]^	47 F	Abdominal pain, weakness, emaciation	No	EUS-FNA
2018	Gebreselassi-e^[29]^	53 F	Weakness, vomiting, abdominal pain	No	EUS-FNA
2018	Lucassen^[30]^	60 F	Abdominal pain, confusion	No	Lymph node biopsy
2020	Ibrahim^[31]^	41 F	Abdominal pain, fatigue, uveitis	No	Skin biopsy
2021	Cantalejo Díaz^[32]^	78 F	Abdominal pain, anorexia, nausea and vomiting, emaciation	No	Abdominal biopsy
2021	Basnet^[33]^	43 M	Abdominal pain, night sweats, weight loss	No	Inguinal lymph node biopsy
2022	Favors^[34]^	50 M	Abdominal pain, back pain, weight loss	No	Pancreaticoduodenect-omy

CT = computed tomography, EUS = endoscopic ultrasound scan, F = female, FNA = fine needle aspiration, M = male.

**Table 2:** Pancreatic sarcoidosis without digestive symptoms.

Year	Author	Patient age/sex	Symptomatic	History of sarcoidosis	Diagnosis/treatment
2011	Delgado-Bolton^[35]^	78 F	asymptomatic	No	biopsy
2013	Yamaguchi^[36]^	80 F	back pain	No	Distal pancreatectomy + splenectomy
2013	Cook^[37]^	60 F	asymptomatic	No	Whipple procedure
2015	Farhat^[38]^	77 F	uveitis	No	biopsy
2017	Matsuura^[39]^	78 F	cough	No	EUS-FNA
2017	Azemoto^[40]^	55 F	asymptomatic	Yes	EUS-FNA
2020	Chedid^[4]^	47 M	asymptomatic	Yes	Distal pancreatectomy
2020	Takeda^[41]^	52 M	asymptomatic	No	EUS-FNA
2023	Chatterjee^[42]^	54 M	asymptomatic	Yes	EUS-FNA

EUS = endoscopic ultrasound scan, F = female, FNA = fine needle aspiration, M = male.

Sarcoidosis is categorized into 2 distinct types: self-limiting and chronic persistent. The self-limited type is characterized by its ability to spontaneously resolve without the need for treatment, while the chronic persistent type is distinguished by its more extensive systemic involvement and the potential for frequent relapses, necessitating therapeutic intervention.^[44]^ The fundamental approach to the management of sarcoidosis entails the administration of immunosuppressive therapy, encompassing corticosteroids, hydroxychloroquine, steroid-sparing agents (methotrexate, azathioprine, leflunomide, mycophenolate mofetil), and anti-tumor necrosis factor-α inhibitors (infliximab and adalimumab).^[1]^ Corticosteroids are a class of pharmaceuticals known for their rapid acting properties. They are employed in various clinical settings, including but not limited to: instances of organ involvement of a critical nature, such as the ocular, nervous system, and cardiac systems; cases of organ involvement accompanied by symptoms; and in the context of symptomatic hypercalcaemia.^[1]^ Consequently, corticosteroids are now widely employed in clinical practice for the treatment of sarcoidosis, as evidenced by the case presented. Following the commencement of oral prednisone acetate, a chest CT scan revealed a reduction in both the pancreatic mass and the mediastinal lymph nodes. Subsequent annual follow-ups demonstrated a substantial decrease in pancreatic, lung and mediastinal lesions, thereby substantiating the diagnosis of sarcoidosis in the pancreatic mass.

## 4. Conclusion

We report a rare case of sarcoidosis involving the pancreas. The patient did not undergo pathological biopsy for definitive diagnosis, but the nodules gradually decreased after oral corticosteroid treatment, suggesting that drug treatment can be used as a diagnostic method in the absence of pathological reports.

## Acknowledgments

The authors express their gratitude to the patient who made this work possible, as well as the professionals and researchers who participated in this study.

## Author contributions

**Writing – original draft:** Rui Li, Nan Gao.

**Investigation:** Qian Liu.

**Writing – review & editing:** Chenghua Zhu, Qiang Du.
